# Relative incidence and individual-level severity of seasonal influenza A H3N2 compared with 2009 pandemic H1N1

**DOI:** 10.1186/s12879-017-2432-7

**Published:** 2017-05-11

**Authors:** Kin On Kwok, Steven Riley, Ranawaka A. P. M. Perera, Vivian W. I. Wei, Peng Wu, Lan Wei, Daniel K. W. Chu, Ian G. Barr, J. S. Malik Peiris, Benjamin J. Cowling

**Affiliations:** 1 JC School of Public Health and Primary Care, Faculty of Medicine, The Chinese University of Hong Kong, Hong Kong, Hong Kong, Special Administrative Region of China; 2 Tanley Ho Centre for Emerging Infectious Diseases, The Chinese University of Hong Kong, Shatin, Hong Kong, Hong Kong, Special Administrative Region of China; 3 WHO Collaborating Centre for Infectious Disease Epidemiology and Control, School of Public Health, Li Ka Shing Faculty of Medicine, The University of Hong Kong, Hong Kong, Hong Kong, Special Administrative Region of China; 4 MRC Centre for Outbreak Analysis and Modelling, Department for Infectious Disease Epidemiology, Imperial College London, London, UK; 5 WHO Collaborating Centre for Reference and Research, Melbourne, VIC Australia; 6 Department of Microbiology and Immunology, University of Melbourne, Melbourne, VIC Australia

**Keywords:** Influenza, Seroepidemiology, Severity, Cohort, Severe outcomes

## Abstract

**Background:**

Two subtypes of influenza A currently circulate in humans: seasonal H3N2 (sH3N2, emerged in 1968) and pandemic H1N1 (pH1N1, emerged in 2009). While the epidemiological characteristics of the initial wave of pH1N1 have been studied in detail, less is known about its infection dynamics during subsequent waves or its severity relative to sH3N2. Even prior to 2009, few data was available to estimate the risk of severe outcomes following infection with one circulating influenza strain relative to another.

**Methods:**

We analyzed antibodies in quadruples of sera from individuals in Hong Kong collected between July 2009 and December 2011, a period that included three distinct influenza virus epidemics. We estimated infection incidence using these assay data and then estimated rates of severe outcomes per infection using population-wide clinical data.

**Results:**

Cumulative incidence of infection was high among children in the first epidemic of pH1N1. There was a change towards the older age group in the age distribution of infections for pH1N1 from the first to the second epidemic, with the age distribution of the second epidemic of pH1N1 more similar to that of sH3N2. We found no serological evidence that individuals were infected in both waves of pH1N1. The risks of excess mortality conditional on infection were higher for sH3N2 than for pH1N1, with age-standardized risk ratios of 2.6 [95% CI: 1.8, 3.7] for all causes and 1.5 [95% CI: 1.0, 2.1] for respiratory causes throughout the study period.

**Conclusions:**

Overall increase in clinical incidence of pH1N1 and higher rates of severity in older adults in post pandemic waves were in line with an age-shift in infection towards the older age groups. The absence of repeated infection is good evidence that waning immunity did not cause the second wave. Despite circulating in humans since 1968, sH3N2 is substantially more severe per infection than the pH1N1 strain. Infection-based estimates of individual-level severity have a role in assessing emerging strains; updating seasonal vaccine components; and optimizing of vaccination programs.

**Electronic supplementary material:**

The online version of this article (doi:10.1186/s12879-017-2432-7) contains supplementary material, which is available to authorized users.

## Background

Pandemics of influenza A occur periodically and are well characterised by waves of increased infection compared with typical inter-pandemic seasons [1], often causing increased morbidity and mortality [2–4]. However, the epidemiological characteristics of the period immediately following a pandemic are less well understood. Since the emergence of the novel influenza A pH1N1 strain in 2009 (pH1N1), subsequent waves of infection have exhibited two intriguing characteristics: they have generated epidemics of similar size to the initial waves in some countries [5], despite no apparent antigenic change; and the distribution of clinical cases was skewed towards the older age groups [6]. Multiple waves with an upward age-shift in cases have also been described for previous pandemics [7].

Widely varying levels of testing over time and changes in the propensity of individuals to seek medical attention make the assessment of influenza severity a complex problem [8, 9]. For example, in 2009, pre-existing surveillance systems were often modified in short-notice in response to rapidly evolving policy requirements and public demand. Therefore, population-based serological studies were widely recognized as important tools to describe patterns of infection, rather than cases [10]. In particular, serological studies were used to confirm that differences in numbers of cases of adults compared with children were being driven by differences in infection but not by differences in pathogenicity [11, 12].

The individual-level severity associated with specific influenza strains is a determinant of the impact of an epidemic, and can be measured in a number of ways. While the risk of mortality among laboratory-confirmed cases was used during and after the 2009 pandemic, it has been shown that this metric varies over many orders of magnitude and is not a suitable measure of severity [13]. Instead, we have proposed the infection fatality risk, the risk of mortality among persons infected with the virus, as a stable and comparable measure of severity [13–15].

Here, we present results from an ongoing longitudinal serological study [14, 16, 17] and population surveillance data, with the objectives of estimating the incidence of pH1N1 and sH3N2 virus infections in Hong Kong from 2009 to 2011, and characterizing the relative virulence of the two currently circulating human strains of influenza A by comparing their respective excess all-cause deaths, excess respiratory deaths and excess respiratory hospitalizations.

## Methods

We first used a longitudinal community-based serological study to estimate age-specific incidence for the different subtypes between rounds of the study. We then made population-wide estimates of excess hospitalization and death so as to estimate the risk of severe events per infection between each round of the study.

**Fig. 1:**
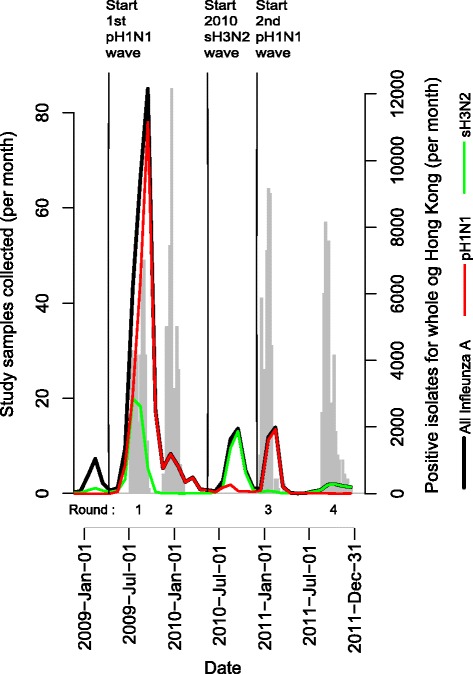
Comparison between our study timeline and community epidemics. Timing of study rounds and laboratory detections of pH1N1 and sH3N2 viruses in Hong Kong from 2009 to 2011. The left y-axis applies to the grey bars, the frequency of sample recruitment by week for four study rounds, with the number of each recruitment round of recruitment indicated below the y-axis. Community epidemics were proxied by the product of weekly proportion of ILI cases among all GP consultations and the weekly proportion of positive subtype-specific test results for all influenza A strain (*black*), pH1N1 (*red*) and sH3N2 (*green*)

In sub-tropical regions, influenza incidence is less predictable than in temperate climates. Therefore, it was not possible to perform the typical design of pre- and post-season sampling. Also, in the period immediately after the pandemic, the timing of local epidemics was especially difficult to predict. Here, we estimate serological incidence and severity between different rounds of the study – not always sequential rounds, depending on the timing of the strain-specific epidemic we were interested in: the timing of the four rounds of the study relative to the epidemics allows us to make inference on the infection attack rate for specific epidemics (Fig. 1).

### Study design

We recruited individuals in Hong Kong via random-digit dialing of household landlines. The study was initiated with the first recruitment round (Round I) between 4 July 2009 and 28 September 2009 (Fig. 1). Details of the initial recruitment have been described in [14]. An individual was recruited during the phone call and was asked to attend the study clinic to answer a questionnaire and to provide a 5 ml serum sample. The responding individual was also asked to invite other eligible members within the household including children. Eligible participants included Hong Kong residents ≥2 years of age. Following Round I, we invited participants to return to the clinic during three subsequent periods: 11 November 2009–6 February 2010 (Round 2), 13 December 2010–19 March 2011 (Round 3) and 24 August 2011–19 December 2011 (Round 4). Participants who provided serum specimens were compensated with HKD100 (~US$13) in the form of supermarket vouchers (adults) or book tokens (children). Ethics approval was obtained from the Institutional Review Board of the University of Hong Kong.

### Outcome measures

The primary outcome measure is laboratory-confirmed influenza infection determined by seroconversion between paired sera, which is defined as a four-fold or greater rise in antibody titres against the two tested strains with the titre value of the latter specimens being at least 1:20. When we reported proportions seroconverting, we state the time period under consideration (for example, proportions of seroconversion between rounds 2 and 3) and we always use the same denominator: we do not remove those who seroconverted between rounds 1 and 2 when we report the proportion of seroconversion between rounds 3 and 4. Individuals who reported vaccination were removed from the denominator for the calculation of all proportions of seroconversion. Because there were clear waves of infection for the different subtypes in Hong Kong during the period of the study, we chose to report proportions seroconverting and state clearly the rounds of the study that formed the start and end of the pair for each outcome (Fig. 1).

### Laboratory Methods

Serum specimens were transferred to the laboratory at 4 °C and frozen to −70 °C prior to testing. Serum samples from each individual were tested in parallel by hemagglutination inhibition (HI) assays against the prevalent A/California/7/2009-like and A/Perth/16/2009-like viruses using standard methods as previously described [14]. The serum specimens were tested in serial doubling dilutions from an initial dilution of 1:10.

### Population surveillance

A sentinel surveillance network for influenza-like illnesses (ILI) was established in 1998 in Hong Kong, and currently provides information on the weekly number of influenza-like illnesses (ILI) and the total weekly number of consultations among a network of approximately 50 general practitioners, allowing estimation of ILI rates in the population, aggregated for all ages [18]. The Public Health Laboratory Services Branch in the Centre for Health Protection conduct laboratory testing of specimens provided by the ILI network and local hospitals for surveillance and diagnostic purposes. We obtained data on the weekly number of influenza-positive specimens by type and subtype, and the weekly number of specimens tested [19, 20].

Three major influenza A epidemics were detected in Hong Kong from 2009 to 2011 (Fig. 1). Despite the early onset of the 2009 pandemic, either by chance or because of aggressive interventions [21], there was very little transmission of influenza A virus between April 2009 and mid-August 2009. Since mid-August 2009, the time point at which students in Hong Kong resumed schools, the main pandemic wave of pH1N1 began to take off. The pH1N1 strain appeared to outcompete the sH3N2 strain such that the remainder of the winter 2009/10 wave of infection was dominated by pH1N1. The main pandemic wave lasted until January 2010. An epidemic of sH3N2 infections began in August 2010 till October 2010 (2010 sH3N2 wave), and was followed by the second wave of pH1N1 at the beginning of 2011 (2011 pH1N1 wave). These three identified community epidemics provided context for the current study.

### Statistical analysis

We compared our study samples and the 2009 Hong Kong population with the Cochran effect size. Chi-square tests or Fisher’s exact tests were used to compare the observed proportion of subjects among vaccinated and unvaccinated subjects under different characteristics. Cumulative incidence of each epidemic is approximated by the proportion of seroconversion between study rounds and the corresponding 95% confidence interval is calculated by the binomial exact method.

Age-specific respiratory hospitalizations, all-cause mortality rates and cause-specific mortality rates in Hong Kong from 1998 to 2011 were estimated with multiple linear regression models. The models allowed for activities of different types/subtypes of influenza viruses, that of respiratory syncytial viruses, environmental temperature, absolute humidity, and temporal trends in mortality rates. Influenza-associated excess hospitalizations and mortality during the epidemics of interest were estimated as the difference between the estimates in the presence and those in the absence of the activity of the concerned influenza virus [19]. The corresponding dataset can be found in Additional file 1: Dataset S2.

## Results

Across all four rounds of recruitment, we obtained sera from at least 1 individual in 1211 households. The total number of people living in those households was 3760 from which we obtained accurate age data on 1381 (37%). From these, we obtained a quadruple set of sera from 420 individuals (Additional file 2: Dataset S1). Table 1 summarizes the baseline characteristics of these 420 participants. Our study samples were biased towards the middle-age groups aged 45–64 while individuals aged 44 or below were under-sampled, compared to the 2009 Hong Kong population. Our samples were similar to the population in terms of sex (ES = 0.13) and living district (ES = 0.08).

**Table 1:** Characteristics of the study cohort stratified by vaccination status and compared with the 2009 government data

	Hong Kong Population(Total = 6912020)^a^		Overall(Total = 420)		ES^b^	Vaccinated^c^(Total = 113)		Unvaccinated^c^(Total = 306)		P-value^d^
	n	%	n	%		n	%	n	%	
Age Group (years)										
2 -18	1129960	16.3	43	10.2	0.50	11	9.7	31	10.1	1.00
19-44	2796560	40.5	101	24.0		19	16.8	82	26.8	0.05
45-64	2092000	30.3	221	52.6		44	38.9	177	57.8	0.00
65+	893500	12.9	55	13.1		39	34.5	16	5.2	0.00
Sex										
Female	3663660	53.0	249	59.3	0.13	62	54.9	186	60.8	0.33
Male	3248360	47.0	171	40.7		51	45.1	120	39.2	0.33
Working Status										
Indivdulas aged 15 or above^e^										
Unemployed/Home-makers/Retired/Others	2041100	29.5	200	47.6	0.43	55	48.7	145	47.4	0.90
Employed populations	3479800	50.3	158	37.6		40	35.4	118	38.6	0.63
Students	535800	7.8	38	9.0		12	10.6	26	8.5	0.63
Indivdulas aged 2-15	855320	12.4	24	5.7		6	5.3	17	5.6	1.00
Education Attainment^f^										
No schooling / Pre-primary	-	-	5	1.2	-	1	0.9	4	1.3	1.00
Primary	-	-	65	15.5		27	23.9	38	12.4	0.01
Secondary	-	-	197	46.9		39	34.5	158	51.6	0.00
Drploma/Certiricate/Sub-degree/Craft-level	-	-	27	6.4		6	5.3	21	6.9	0.73
Degree or above	-	-	62	14.8		22	19.5	40	13.1	0.14
Studying (full-time)	-	-	62	14.8		18	15.9	43	14.1	0.74
Missing	-	-	2	0.5	-	0	0.0	2	0.7	-
Living District^g^										
Hong Kong Island	1276074	18.5	77	18.3	0.08	16	14.2	61	19.9	0.23
Kowloon	2036784	29.5	134	31.9		39	34.5	95	31.0	0.58
New Territories	3597089	52.0	200	47.6		56	49.6	143	46.7	0.69
Board vessels	2073	0.0	0	0.0		0	0.0	0	0.0	1.00
Missing	0	0.0	9	2.1	-	2	1.8	7	2.3	-

^a^2009 population aged 2 or above

^b^ES refers to effect size calculated by Cochran Q test

^c^There is one subject without vaccination status

^d^P-values are calculated by chi-square tests or Fisher's exact tests

^e^Only individuals aged 15 or above, lived not on Board vessels and not admitted to hospitals were included in the 2009 government data

^f^Education attainment were only obtained for non full-time students while that of census also included full-time students

^g^Uniform age distribution within the age group 0-4 and even spatial distribution among districts were assumed for the 2009 government data

Of the 420 individuals considered here, 113 reported receipt of one of the three vaccinations used during the study period: 2009/10 trivalent seasonal vaccine, 2009 monovalent pandemic vaccine or 2010/11 trivalent seasonal vaccine. The proportion of elderly (aged 65 or above) among vaccinated individuals was significantly higher than that among unvaccinated (*p*-value = 0.00). On the contrary, that of young adults (aged 19–64) was significantly lower among the vaccinated group than the unvaccinated group (*p*-value = 0.05 and 0.00 respectively). Lower education attainment (primary/secondary) was also associated with higher proportion of receipt of seasonal vaccination (*p*-value = 0.01 and 0.00 respectively). Other comparisons of vaccinated subjects and unvaccinated subjects were also described in Table 1.

Our study period covered a period of three community epidemics (Fig. 1) of influenza A virus in Hong Kong from 2009 to 2011. Although the rounds of study did not precisely bracket the epidemics, we were able to make good inferences for the infection attack rate for each wave. Despite the slight non-bracketing of Rounds 1 and 2 to the 2009 pandemic (denoted as the main pandemic wave) as describe in [14], our study samples from these two rounds were able to capture the peak period of this wave. Thus estimates from study Rounds 1 and 2 are used to draw inference on the main pandemic wave. The 2010 sH3N2 wave was bracketed by study Rounds 2 and 3 whereas the 2011 pH1N1 wave was bracketed by study Rounds 2 and 4.

**Fig. 2:**
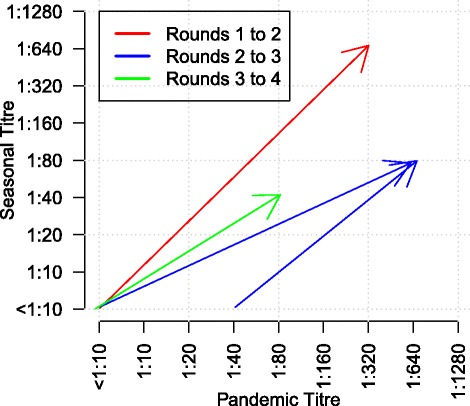
Transition of five HI titre pairs that exhibited a four-fold rise or greater in antibody titres against sH3N2 and pH1N1. The colour legend indicates the study rounds from which the sera pair was drawn. The start of each arrow represents titre values of the samples taken from the former study round and the end of the arrow represents that of the samples from the latter round. Titre values against pH1N1 is on x-axis and that against sH3N2 is on the y-axis. A small amount of noise was added to distinguish lines that start or end at the same coordinate

We did not find evidence that waning of immunity was an important feature of these epidemics, We observed a total of 22 seroconversions to pH1N1 between Rounds 1 and 2 and 66 infections between Rounds 2 and 4. There was no overlap between these groups. Although there was a suggestion that seroconversion to sH3N2 between Rounds 2 and 3 was protective against infection with pH1N1 between Rounds 2 and 4, this pattern was not statistically significant because the 95% confidence interval for nominal overall proportions of seroconversion did not overlap (Table 2). Across the entire study period, we recorded only 4 instances in which an individual seroconverted to more than one strain in a single period (Fig. 2). These may have arisen from unreported vaccination, co-infections or sequential infections with different viruses.

**Table 2:** Numbers and proportion^1^ of seroconversions between study rounds for seasonal H3N2 and pandemic H1N1 among unvaccinated participants

Subtype	Rounds^a^	Nominal overall^b^(n = 306)	2-18 (n = 31)	19-44 (n = 82)	45-64 (n = 177)	65+ (n = 16)
H1N1	1,2	29 0.09 (0.07,0.13)	10 0.32 (0.19,0.51)	7 0.09 (0.04,0.17)	5 0.03 (0.01,0.06)	0 0.00 (0.00,0.21)
	2,4	68 0.22 (0.18,0.27)	11 0.35 (0.22,0.55)	18 0.22 (0.15,0.32)	35 0.20 (0.15,0.26)	2 0.12 (0.04,0.38)
H3N2	2,3	23 0.08 (0.05,0.11)	2 0.06 (0.02,0.21)	4 0.05 (0.02,0.12)	16 0.09 (0.06,0.14)	2 0.12 (0.04,0.38)

^a^Rounds of study providing the pair of samples on which seroconversion status was based

^b^Nominal overall represents the expected overall number of infections if the age distribution of the study were the same as the age distribution of Hong Kong

**Fig. 3:**
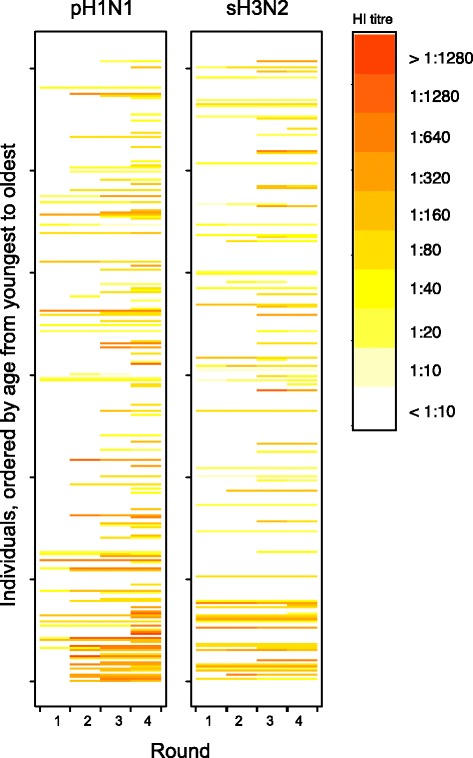
Antibody titre values of pH1N1 and sH3N2. Each row of pixels represents an individual and each column of pixels represents study rounds. Titer values are indicated in the color legend

**Fig. 4:**
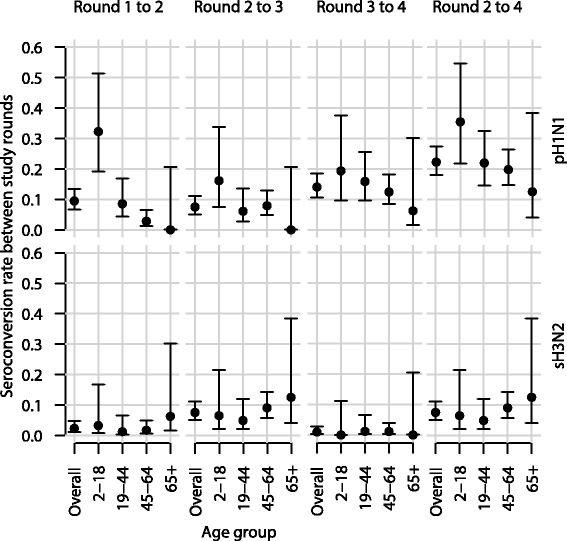
Proportions of individuals with seroconversion. Overall and age-specific seroconversion proportions between study rounds (*column*) against different influenza A strains (*row*). Overall proportions are estimated by the proportion of seroconversoins that would have been obtained in each age group had the age distribution of study participant been perfectly representative to that of the general population. Vertical bars represent the 95% confidence intervals assuming the binomial distribution

Although the timing of study rounds did not produce clear pre- and post-season sera for epidemic, the interval between some pairs within our study was clearly dominated by one strain (Fig. 1). We observed a significant shift in the age profile of pH1N1 seroconversions towards older participants between the period bracketed by Rounds 1 and 2 compared with the period bracketed by Rounds 2 and 4 (*p*-value = 0.01 for Pearsons chi squared test; Figs. 3 and 4). The change can be seen in age-specific rates of seroconversion (Fig. 4, Table 2). Cumulative incidence in participants aged 2–18 was approximately constant with 10 (out of 31) infections between Rounds 1 and 2 (0.32 [0.19, 0.51]) compared with 11 (out of 31) infections between Rounds 2 and 4 (0.35 [0.22, 0.55]). Conversely, the cumulative incidence in participants aged 45–64 increased from 0.03 [0.01, 0.06] between Rounds 1 and 2 to 0.20 [0.15, 0.26] between Rounds 2 and 4. Although there was an increase in the number of seroconversions among participants aged 19–44, this was not statistically significant.

We estimated that infection incidence of the main pandemic wave was approximately half that of the 2011 pH1N1 wave. Age-standardized cumulative incidence was 0.09 [0.07, 0.13] between Rounds 1 and 2 for pH1N1 compared with 0.22 [0.18, 0.27] between Rounds 2 and 4 for pH1N1. The intervening 2010 sH3N2 wave generated milder level of infection compared with the 2011 pH1N1 wave, with an overall age standardized cumulative incidence of 0.08 [0.05, 0.11]. This is contrary to the similar epidemic profile based on laboratory isolation data (Fig. 1) and there should be no obvious changes in testing practices in such a short period of time.

Despite the difference in amplitude, the age distribution of infections in the 2011 pH1N1 wave was not significantly different from that of the sH3N2 epidemic (*p*-value = 0.26 for Pearsons chi squared test), with similar elevated incidence among participants aged 45–64 (Fig. 4). Among these unvaccinated individuals from whom we obtained a quadruple set of sera, there were 4 observed infections among participants aged 65 or above (Table 2). Two of the infection events occurred between Rounds 2 and 3, while the other two occurred between Rounds 2 and 4, with age-specific cumulative incidence of both pH1N1 and sH3N2 infection being 0.12 [0.04, 0.38].

**Fig. 5:**
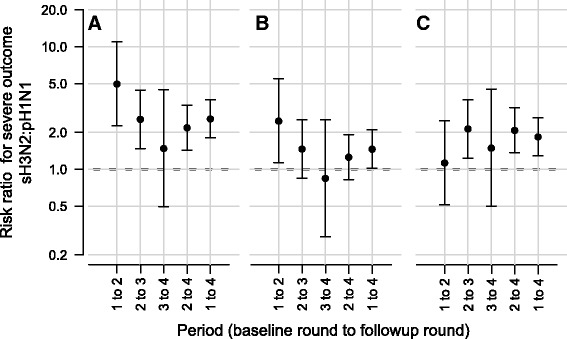
Virulence of sH3N2 compared to pH1N1 in terms of (**a**) excess all-cause death, (**b**) an excess respiratory death and (**c**) excess respiratory hospitalization. It is expressed as the ratio of the estimated risk of adverse events caused by sH3N2 infection relative to pH1N1. Values above one indicate higher estimated risk for sH3N2. Confidence intervals are calculated using a score confidence interval [32] as implemented in the riskscoreci function in the R package PropCIs

We characterized the virulence of these strains with excess all-cause deaths, excess respiratory deaths, and excess respiratory hospitalizations [19]. Absolute risks of severe outcomes per infection were generally higher for sH3N2 than for pH1N1 (Table 3). Therefore, we estimated the ratio of risks of severe outcomes per infection of sH3N2 to that of pH1N1 (Fig. 5). These data suggest that infection with sH3N2 was approximately twice more likely to result in a severe outcome than infection with pH1N1 (Fig. 5). Throughout the study period, the risk of excess all-cause mortality, excess respiratory mortality and excess pneumonia and influenza hospitalization for sH3N2 relative to pH1N1 was 2.6 [1.8, 3.7], 1.5 [1.0, 2.1], and 1.8 [1.3, 2.6] respectively.

**Table 3:** Estimated absolute risks of severe outcomes per infection

Outcome	Strain	Rounds	Infections^a^	Estimated severe outcomes^b^	Risk per 10,000 infections (Binomial 95% CI)^c^
Excess all-cause deaths	A(H1N1)pdm09	1 to 2	655000	61	0.93 (0.66 , 1.31)
		2 to 3	520000	160	3.08 (2.09 , 4.55)
		3 to 4	971000	359	3.70 (2.81 , 4.88)
	A(H3N2)	1 to 2	158000	73	4.62 (2.27 , 9.30)
		2 to 3	520000	409	7.87 (5.35 , 11.6)
		3 to 4	67800	37	5.46 (1.89 , 14.9)
Excess respiratory deaths	A(H1N1)pdm09	1 to 2	655,000	57	0.87 (0.62 , 1.23)
		2 to 3	520,000	130	2.50 (1.70 , 3.70)
		3 to 4	971000	289	2.98 (2.27 , 3.93)
	A(H3N2)	1 to 2	158000	34	2.15 (1.06 , 4.33)
		2 to 3	520000	190	3.66 (2.48 , 5.40)
		3 to 4	67800	17	2.51 (0.87 , 6.83)
Excess respiratory hospitalisations	A(H1N1)pdm09	1 to 2	655000	3470	53.0 (37.7 , 74.8)
		2 to 3	520000	2480	47.7 (32.4 , 70.5)
		3 to 4	971000	4590	47.2 (36.0 , 62.4)
	A(H3N2)	1 to 2	158000	942	59.6 (29.3 , 120)
		2 to 3	520000	5300	102 (69.3 , 151)
		3 to 4	67800	476	70.2 (24.3 , 191)

^a^Estimated cumulative number of seroconversions between study rounds, adjusted for the difference in the age distribution between study participants and the overall Hong Kong population. For each age group i in our study data (i = 1,2,3,4), which corresponds to age groups (2-18, 19-44,45-64,65+ ), we define A*i*=Age group specific proportion of four fold rise in our study data x sample size of this study x Proportion of population in this age group

Then, number of infections can be calculated with the following formula

$$ Hong\  Kong\  population\  X\frac{\sum_{i=1}^4\mathrm{Ai}}{our\  study\  sample\  size} $$

^b^Estimated excess rate of adverse outcomes. Point estimates calculated as in [26]

^c^Binomial confidence bounds reflecting uncertainty in the number of infections, rather than the number of adverse outcomes. See Discussion

## Discussion

We used a set of quadruple sera from a longitudinal serologic study in Hong Kong to provide community-based estimates of infection for the three influenza virus epidemics, namely the 2009 pH1N1 wave from July 2009 to January 2010, the 2010 sH3N2 wave from August 2010 to October 2010, and the 2011 pH1N1 wave from January 2011 to February 2011 (Table 2). For the two pH1N1 waves (i.e. the 2009 pH1N1 wave and the 2011 pH1N1 wave), proportion of adults aged 19 or above infected in the latter wave was much higher than that in the former wave. Hong Kong is a developed subtropical city with a mobile and dense population, incidence of influenza virus infections and transmission dynamics may differ from other locations [22–25]. However, our observation here about the age-shift infection pattern towards the older age groups during the latter pandemic wave was also observed in other places [6].

We also observed that individuals infected in the 2009 pH1N1wave were not infected again during the 2011 pH1N1 wave, suggesting infection in prior waves might be protective of infection in latter wave. This also suggests that the 2011 pH1N1 wave was not generated by the waning of immunity or re-infection of individuals infected during the 2009 pandemic wave.

We combined our estimates of cumulative incidence of infection with estimates of excess influenza mortality for each community epidemic to compare the virulence of pH1N1 and sH3N2 viruses [26]. We estimated that the infection fatality risk for sH3N2 was approximately twice as high as the infection fatality risk for pH1N1. Although unable to estimate age-specific virulence ratios, our age-adjusted overall estimate was consistent for multiple severe outcomes and across different influenza seasons.

Our results generalize the severity comparisons among strains from sick individuals to the general population. According to Yang et al. [27], individuals with H1N1pdm09 pneumonia showed higher disease diversity and stronger systematic inflammatory response than their counterparts infected with A (H3N2) pneumonia. On the contrary, here, it was sH3N2 infections which caused more number of excess deaths and excess hospitalizations in the community.

Our study was similar to the Flu Watch study in the UK that estimated strain specific infection fatality rates but was unable to find any significant differences between strains [28]. Despite the relatively small sample size, the key advantage of the present study was that we were able to combine study-derived serological data and population-level measures of severity, rather than relying on severe events observed within the cohort. In addition, the timing between our study rounds and community epidemics was close that the serologic data (from our study) and the severity data (with respect to the community epidemics) were highly consistent over this short period of time. Therefore, even with a relatively small number of quadruple sets of longitudinal sera, we were still well-powered to detect a difference in the per-infection risk of severe outcomes.

Our study has a number of limitations. Our study rounds did not neatly bracket each community epidemic: Round 1 was taken towards the start of the initial prolonged wave of the 2009 pH1N1 pandemic and Round 2 was close to the end of that period. However, our focus here was on relative severity rather than the estimation of the absolute attack rate for the main pandemic wave, which we have addressed previously by synthesizing microneutralization results and surveillance data [14]. On the other hand, Rounds 2 and 4 bracketed the 2011 pH1N1 wave but the interval between them was long that it might include some infection events from the end of the 2009 pH1N1 wave. The prolonged gap between consecutive sera collection may underestimate the cumulative incidence of infections due to waning in antibody titers over time back to baseline levels [29], although we found no evidence of waning. Also, our confidence bounds for rates of excess adverse events and ratios of excess adverse events (Table 3) reflected only uncertainty in rates of infection but not the uncertainty from the regression analysis in which the number of excess events was calculated. Therefore, the confidence bounds may not reflect the true degree of uncertainty.

Variation in the timing of the study rounds may have led to differential recall bias about vaccination. Given that our outcome was serological, any systematic bias in recall of vaccination would have influenced both our numerator and denominator for estimated proportions of seroconversion. However, during this period immediately after the pandemic, awareness of vaccination was high in Hong Kong and there was at most only a full year between rounds of the study. Therefore, we feel confident that recall of vaccination status will have been at least as good in our study to other similar cohort studies.

We excluded vaccinated individuals and therefore assume implicitly that they do not contribute substantially to the epidemiology of these outbreaks. In the absence of any validated method for distinguishing titer rises due to vaccination from titer rises due to infection, this was a necessary simplification. However, the resulting description of the relative severity of the different strains may be affected by patterns of vaccination from age group to age group and from year to year.

As with any similar study, we cannot be certain that our study population is representative of the population for the process we are most interested in, namely, influenza infection. However, when the cohort was setup, we did have the opportunity to compare two types of household -those from genuinely random digit dialing and those who had participated in a prior questionnaire-only study before also joining this study [14]. Essentially, the latter group was even more likely to participate In a study of this type than the main group. We found no difference in outcomes between those two types when the study was first set up. Since then, new households have been recruited via random-digit dialing.

Another limitation was that, infection may sometimes only trigger low boosting, leading to underestimation of infection incidence [29] . Also, our study is not representative to the population (Table 1) and overall recruitment rates were low [14]. However, via a number of internal consistency checks [14] and after comparing with cross-sectional data from an entirely separate study in the same population at the same time [30], and with other studies [11], we found no evidence of systematic bias in our cohort with respect to influenza infection (other than by age for which we adjust our results). In addition, as discussed above, the key strength of the present study, which must be weighed against its relatively small size, is its consistency and duration.

In short, our findings on the incidence and virulence of pH1N1 and sH3N2 viruses provide important insights into human influenza ecology. Following the first wave of pH1N1 wave with high incidence of infection in children, further epidemics in the absence of antigenic drift were not expected [31]. However in early 2011, Hong Kong experienced a second wave of pH1N1 with substantial impact without virological evidence of antigenic drift [26]. There was a substantial shift in infections away from children towards middle-aged adults between the first and second waves of pH1N1 in Hong Kong (Table 2, Fig. 4). The age distribution of infections in the second wave of pH1N1 was similar to that of the 2010 sH3N2 wave which intertwined the two pH1N1 waves, implying that middle-aged adults may be more important for influenza A transmission during non-pandemic periods than has been assumed previously.

## Conclusions

We have demonstrated additional value from long-term seroepidemiological studies. By providing reliable data on incidence of influenza virus infections and combining those data with analysis of excess mortality, it is possible to generate robust estimates of the virulence of one strain relative to another. Continuation of this study, and replication in other locations, would provide further information on the dynamics of annual influenza epidemics, and would also allow capacity to be maintained for similar studies during the next influenza pandemic. Such studies could be important in providing ongoing accurate measures of incidence and hence virulence, which is critical for monitoring the evolution of human influenza strains in the period up to the next pandemic.

## Additional files

Additional file 1: Dataset S2 Population-level surveillance data. All weekly records used to calculate excess hospitalisations and deaths are contained in this comma separated file. (CSV 35 kb)

Additional file 2: Dataset S1Individual-level data from the serological survey. All records and fields from the cohort study used in the main analysis are contained in this comma separated file. (CSV 48 kb)

## Abbreviations

ILI: Influenza-like-illness
pH1N1: Pandemic 2009 influenza A H1N1
R1: Round 1
sH3N2: Seasonal influenza A H3N2
